# Iodo­durene

**DOI:** 10.1107/S1600536812046557

**Published:** 2012-11-24

**Authors:** Noudjoud Hamdouni, Ouarda Brihi, Mohamed Larbi Medjroubi, Jean Meinnel, Ali Boudjada

**Affiliations:** a Laboratoire de Cristallographie, Département de Physique, Université Mentouri-Constantine, 25000 Constantine, Algeria; b UMR 6226 CNRS–Université Rennes 1, Sciences Chimiques de Rennes, Equipe Matière Condensée et Systèmes Electroactifs, 263 Avenue du Général Leclerc, F-35042 Rennes, France

## Abstract

The title compound (systematic name: 1-iodo-2,3,5,6-tetra­methyl­benzene), C_10_H_13_I, crystallizes in the chiral space group *P*2_1_2_1_2_1_. The I atom is displaced by 0.1003 (5) Å from the mean plane of the ten C atoms [maximum deviation = 0.018 (6) Å]. In the crystal, there are no significant inter­molecular inter­actions present.

## Related literature

For the crystal structure of bromo­durene, see: Charbonneau *et al.* (1964 ▶, 1965 ▶). For the physical properties of mono-halogen­ated derivatives of durene, see: Balcou *et al.* (1965 ▶). For standard bond lengths in similar compounds, see: Allen (2002 ▶); Hope *et al.* (1970 ▶).
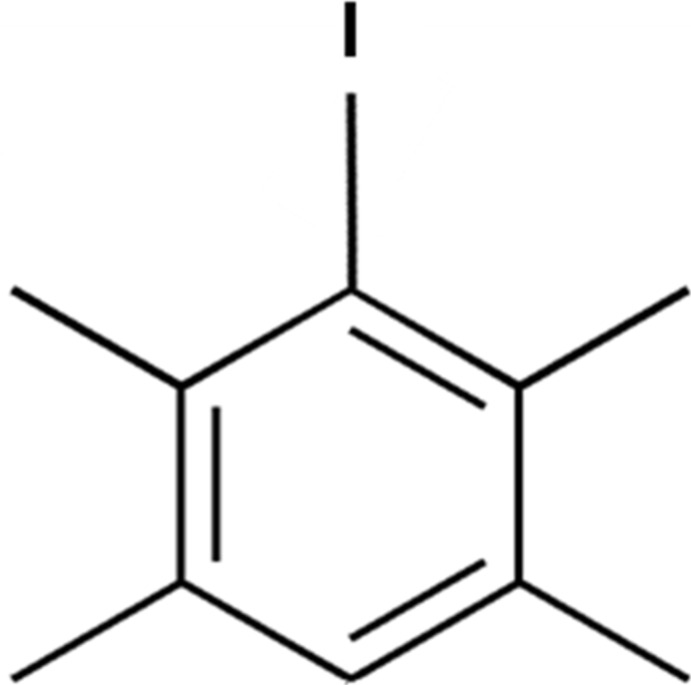


## Experimental

### 

#### Crystal data

C_10_H_13_I
*M* *_r_* = 260.11
Orthorhombic, 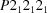

*a* = 5.5099 (3) Å
*b* = 11.8839 (5) Å
*c* = 15.1704 (6) Å
*V* = 993.34 (8) Å^3^
*Z* = 4
Mo *K*α radiation
μ = 3.16 mm^−1^
*T* = 293 K
0.10 × 0.05 × 0.04 mm

#### Data collection

Nonius KappaCCD diffractometer
Absorption correction: multi-scan (*SADABS*; Sheldrick, 1996 ▶) *T*_min_ = 0.140, *T*_max_ = 0.193
26027 measured reflections
2271 independent reflections
2036 reflections with *I* > 3σ(*I*)
*R*_int_ = 0.047

#### Refinement

*R*[*F* ^2^ > 2σ(*F* ^2^)] = 0.035
*wR*(*F* ^2^) = 0.039
*S* = 1.04
3272 reflections
101 parameters
36 restraints
H-atom parameters constrained
Δρ_max_ = 0.84 e Å^−3^
Δρ_min_ = −0.71 e Å^−3^
Absolute structure: Flack (1983 ▶), 925 Friedal pairs
Flack parameter: −0.03 (4)

### 

Data collection: *COLLECT* (Nonius, 2001 ▶); cell refinement: *DIRAX/LSQ* (Duisenberg *et al.*, 2003 ▶); data reduction: *EVALCCD* (Duisenberg *et al.*, 2003 ▶); program(s) used to solve structure: *SIR2002* (Burla *et al.*, 2005 ▶); program(s) used to refine structure: *CRYSTALS* (Betteridge *et al.*, 2003 ▶); molecular graphics: *CAMERON* (Watkin *et al.*, 1996 ▶) and *DIAMOND* (Brandenburg, 2012 ▶); software used to prepare material for publication: *CRYSTALS*.

## Supplementary Material

Crystal structure: contains datablock(s) global, I. DOI: 10.1107/S1600536812046557/su2521sup1.cif

Structure factors: contains datablock(s) I. DOI: 10.1107/S1600536812046557/su2521Isup2.hkl

Supplementary material file. DOI: 10.1107/S1600536812046557/su2521Isup3.cml

Additional supplementary materials:  crystallographic information; 3D view; checkCIF report

## Figures and Tables

**Fig. 1.**
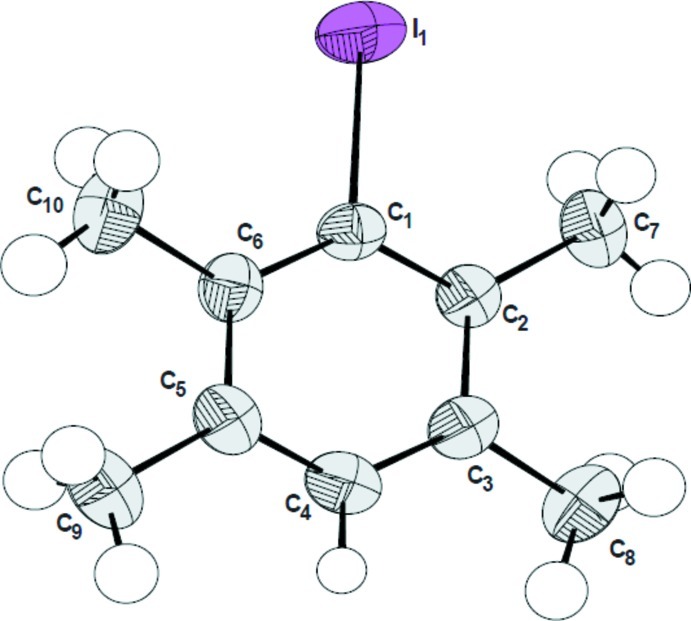
The molecular structure of the title molecule, with atom numbering. The displacement ellipsoids are drawn at the 50% probability level.

**Fig. 2.**
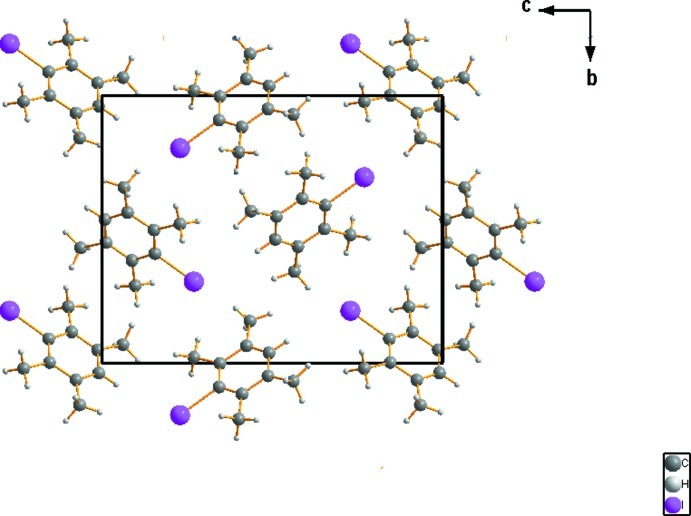
A view along the a axis of the crystal packing of the title compound.

**Fig. 3.**
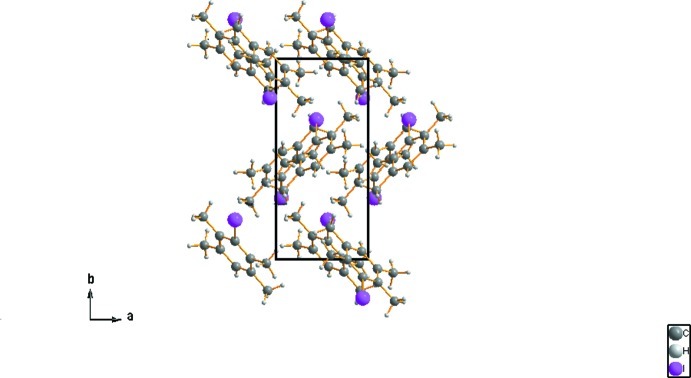
A view along the c axis of the crystal packing of the title compound.

## supplementary crystallographic information

### Comment

Substituted benzene compounds are generally liquid at room temperature so relatively few crystal structure analyses of such compounds have been reported. We have synthesized three mono-halogenated products of duren; chloro-, bromo- and iododurene. These derivatives are solid at 293 K and their dielectric behavior depends on the nature of the substituent, and in particular of its mass and volume. Contrary to chlorodurene the title iododurene in the solid phase has a quite low permeability equal to only 2.6, which changes abruptly at fusion to a value of ca. 4 (Balcou *et al.*, 1965). We report herein on its crystal structure.

The molecular structure of the title compound is illustrated in Fig. 1. The molecule is almost planar with the I atom displaced from the mean plane of the nine C atoms [maximum deviation 0.018 (6) Å for the methyl C atoms C7 and C9] by 0.1003 (5) Å. The average endocyclic angles facing the I atom and the methyl groups are 124.98 (10)° and 117.41 (10)°, respectively. The structural study did not reveal any disorder and the average intramolecular bond lengths, [C_ar_-I = 2.139 (3) Å, C_ar_-C_ar_ = 1.400 (5) Å and C_ar_-C_Me_ = 1.498 (6) Å], as well as the average single C_ar_-H bond length [0.940 Å], agree with the distances reported in the literature [Cambridge Structural Database (Allen, 2002); Hope *et al.*, 1970].

In the crystal, the *a* axis is very short, so the molecules stack along this direction (Fig. 2). The best mean plane passing through the carbon and iodine atoms, calculated with the program Molax of *CRYSTALS* (Betteridge *et al.*, 2003), indicated that the angle between the normal to this mean plane is ca. 50.1° relative to the *a* axis, ca. 44.8° relative to the *b* axis and ca. 74.5° relative to the *c* axis (Fig. 3).

The crystals obtained sublimate a little at ambient temperature. Moreover, at room temperature iododurene and the isotypic product bromodurene (Charbonneau *et al.*, 1964, 1965) crystallize in the same orthorhombic space group with fixed orientations of the dipolar grouping not presenting dielectric dispersion. This fact could be interpreted by the importance of the distribution of the masses of the substituents, that inhibit the possibility of molecular reorientation like in chlorodurene (Balcou *et al.*, 1965).

### Experimental

The title compound was synthesized by direct iodination of durene. Equimolecular quantities of iodine and durene were dissolved in pure acetic acid at 343 K. Progressive iodination was obtained by oxidation of durene using drops of pure sulfuric and nitric acids diluted in acetic acid. Total precipitation of iododurene was obtained by dilution with water. Purification was done by chromatography on a silica column and finally precipitation of a solution in chloroform, giving colouress needle-like crystals.

### Refinement

All H atoms were localized in a difference Fourier map. The C-bound H atoms were included in calculated positions and treated as riding atoms: C-H = 0.94 Å for CH H atoms, and 0.95 - 0.97 Å for CH_3_ H atoms, with U_iso_(H) = k × U_eq_(C) where k = 1.5 for CH_3_ H atoms and = 1.2 for other H atoms.

### Crystal data

**Table:**

C_10_H_13_I	*D*_x_ = 1.739 Mg m^−^^3^
*M**_r_* = 260.11	Mo *K*α radiation, λ = 0.71073 Å
Orthorhombic, *P*2_1_2_1_2_1_	Cell parameters from 8232 reflections
*a* = 5.5099 (3) Å	θ = 3.7–27.5°
*b* = 11.8839 (5) Å	µ = 3.16 mm^−^^1^
*c* = 15.1704 (6) Å	*T* = 293 K
*V* = 993.34 (8) Å^3^	Needle, colourless
*Z* = 4	0.10 × 0.05 × 0.04 mm
*F*(000) = 503.986	

### Data collection

**Table:**

Nonius KappaCCD diffractometer	2036 reflections with *I* > 3σ(*I*)
Graphite monochromator	*R*_int_ = 0.047
CCD scans	θ_max_ = 27.5°, θ_min_ = 3.7°
Absorption correction: multi-scan (*SADABS*; Sheldrick, 1996)	*h* = −7→7
*T*_min_ = 0.140, *T*_max_ = 0.193	*k* = −15→15
26027 measured reflections	*l* = −19→19
2271 independent reflections	

### Refinement

**Table:**

Refinement on *F*	Secondary atom site location: difference Fourier map
Least-squares matrix: full	Hydrogen site location: inferred from neighbouring sites
*R*[*F*^2^ > 2σ(*F*^2^)] = 0.035	H-atom parameters constrained
*wR*(*F*^2^) = 0.039	W = [*w*eight]*[1-(delta*F*/6*sigma*F*)^2^]^2^
*S* = 1.04	(Δ/σ)_max_ < 0.001
3272 reflections	Δρ_max_ = 0.84 e Å^−^^3^
101 parameters	Δρ_min_ = −0.71 e Å^−^^3^
36 restraints	Absolute structure: Flack (1983), 925 Friedal pairs
Primary atom site location: structure-invariant direct methods	Flack parameter: −0.03 (4)

### Special details

**Table:**

Geometry. Bond distances, angles etc. have been calculated using the rounded fractional coordinates. All su's are estimated from the variances of the (full) variance-covariance matrix. The cell esds are taken into account in the estimation of distances, angles and torsion angles
Refinement. Refinement of *F*^2^ against ALL reflections. The weighted *R*-factor *wR* and goodness of fit *S* are based on *F*^2^, conventional *R*-factors *R* are based on *F*, with *F* set to zero for negative *F*^2^. The threshold expression of *F*^2^ > σ(*F*^2^) is used only for calculating *R*-factors(gt) *etc*. and is not relevant to the choice of reflections for refinement. *R*-factors based on *F*^2^ are statistically about twice as large as those based on *F*, and *R*- factors based on ALL data will be even larger.

### Fractional atomic coordinates and isotropic or equivalent isotropic displacement parameters (Å^2^)

**Table:**

	*x*	*y*	*z*	*U*_iso_*/*U*_eq_	
I1	0.93848 (10)	0.80448 (4)	0.27027 (3)	0.0686 (2)	
C1	0.9383 (7)	0.9098 (3)	0.1559 (2)	0.0429 (11)	
C2	0.7711 (8)	0.9981 (4)	0.1533 (3)	0.0473 (16)	
C3	0.7637 (8)	1.0638 (3)	0.0755 (3)	0.0483 (16)	
C4	0.9270 (10)	1.0376 (4)	0.0091 (3)	0.0507 (16)	
C5	1.0976 (8)	0.9513 (4)	0.0123 (2)	0.0460 (14)	
C6	1.1033 (7)	0.8838 (3)	0.0887 (3)	0.0423 (14)	
C7	0.5997 (12)	1.0208 (5)	0.2295 (4)	0.071 (2)	
C8	0.5866 (12)	1.1574 (5)	0.0651 (4)	0.067 (2)	
C9	1.2673 (11)	0.9310 (5)	−0.0622 (4)	0.064 (2)	
C10	1.2821 (11)	0.7894 (5)	0.0980 (3)	0.0597 (19)	
H41	0.92110	1.08070	−0.04290	0.0610*	
H71	0.69240	1.03120	0.28260	0.1071*	
H72	0.50940	1.08740	0.21780	0.1072*	
H73	0.49160	0.95840	0.23690	0.1071*	
H81	0.62710	1.21740	0.10510	0.1008*	
H82	0.59650	1.18410	0.00600	0.1008*	
H83	0.42310	1.13100	0.07630	0.1010*	
H91	1.24630	0.98940	−0.10560	0.0971*	
H92	1.43080	0.93360	−0.04010	0.0970*	
H93	1.23570	0.85840	−0.08810	0.0971*	
H101	1.38670	0.80080	0.14830	0.0892*	
H102	1.38000	0.78630	0.04650	0.0892*	
H103	1.19650	0.71900	0.10410	0.0891*	

### Atomic displacement parameters (Å^2^)

**Table:**

	*U*^11^	*U*^22^	*U*^33^	*U*^12^	*U*^13^	*U*^23^
I1	0.0831 (3)	0.0727 (3)	0.0500 (2)	−0.0082 (2)	−0.0036 (2)	0.0120 (2)
C1	0.046 (2)	0.044 (2)	0.0387 (19)	−0.006 (2)	−0.004 (2)	0.0006 (17)
C2	0.047 (3)	0.044 (2)	0.051 (3)	−0.005 (2)	0.000 (2)	−0.009 (2)
C3	0.046 (3)	0.040 (2)	0.059 (3)	−0.004 (2)	−0.011 (2)	−0.007 (2)
C4	0.055 (3)	0.045 (2)	0.052 (3)	−0.007 (3)	−0.011 (3)	0.0031 (19)
C5	0.047 (3)	0.048 (2)	0.043 (2)	−0.010 (2)	−0.001 (2)	−0.0036 (18)
C6	0.041 (3)	0.041 (2)	0.045 (2)	−0.0034 (18)	−0.0062 (18)	−0.0043 (17)
C7	0.070 (4)	0.071 (3)	0.072 (4)	0.000 (3)	0.013 (4)	−0.021 (3)
C8	0.063 (4)	0.052 (3)	0.087 (4)	0.008 (3)	−0.019 (4)	−0.005 (3)
C9	0.060 (4)	0.077 (4)	0.056 (3)	−0.009 (3)	0.005 (3)	−0.011 (3)
C10	0.056 (3)	0.053 (3)	0.070 (4)	0.011 (3)	−0.006 (3)	−0.008 (3)

### Geometric parameters (Å, º)

**Table:**

I1—C1	2.139 (3)	C7—H71	0.9600
C1—C2	1.397 (6)	C7—H72	0.9500
C1—C6	1.401 (5)	C7—H73	0.9600
C2—C3	1.416 (6)	C8—H81	0.9600
C2—C7	1.517 (8)	C8—H82	0.9500
C3—C4	1.386 (7)	C8—H83	0.9700
C3—C8	1.488 (7)	C9—H91	0.9600
C4—C5	1.392 (7)	C9—H92	0.9600
C5—C6	1.410 (6)	C9—H93	0.9600
C5—C9	1.487 (7)	C10—H101	0.9700
C6—C10	1.500 (7)	C10—H102	0.9500
C4—H41	0.9400	C10—H103	0.9600
			
I1—C1—C2	117.6 (3)	H71—C7—H72	109.00
I1—C1—C6	117.5 (3)	H71—C7—H73	109.00
C2—C1—C6	125.0 (3)	H72—C7—H73	110.00
C1—C2—C3	117.2 (4)	C3—C8—H81	110.00
C1—C2—C7	121.5 (4)	C3—C8—H82	108.00
C3—C2—C7	121.3 (4)	C3—C8—H83	110.00
C2—C3—C4	117.6 (4)	H81—C8—H82	109.00
C2—C3—C8	121.3 (4)	H81—C8—H83	110.00
C4—C3—C8	121.1 (4)	H82—C8—H83	109.00
C3—C4—C5	125.4 (4)	C5—C9—H91	109.00
C4—C5—C6	117.6 (4)	C5—C9—H92	109.00
C4—C5—C9	121.2 (4)	C5—C9—H93	110.00
C6—C5—C9	121.2 (4)	H91—C9—H92	109.00
C1—C6—C5	117.3 (3)	H91—C9—H93	110.00
C1—C6—C10	121.5 (4)	H92—C9—H93	110.00
C5—C6—C10	121.2 (4)	C6—C10—H101	111.00
C3—C4—H41	118.00	C6—C10—H102	109.00
C5—C4—H41	117.00	C6—C10—H103	110.00
C2—C7—H71	109.00	H101—C10—H102	108.00
C2—C7—H72	109.00	H101—C10—H103	110.00
C2—C7—H73	110.00	H102—C10—H103	109.00
			
I1—C1—C2—C3	−176.8 (3)	C7—C2—C3—C4	179.9 (5)
I1—C1—C2—C7	1.5 (6)	C7—C2—C3—C8	−0.3 (7)
C6—C1—C2—C3	2.1 (6)	C2—C3—C4—C5	0.4 (7)
C6—C1—C2—C7	−179.6 (4)	C8—C3—C4—C5	−179.4 (5)
I1—C1—C6—C5	178.1 (3)	C3—C4—C5—C6	0.9 (7)
I1—C1—C6—C10	−3.0 (5)	C3—C4—C5—C9	−179.2 (5)
C2—C1—C6—C5	−0.8 (6)	C4—C5—C6—C1	−0.7 (6)
C2—C1—C6—C10	178.1 (4)	C4—C5—C6—C10	−179.6 (4)
C1—C2—C3—C4	−1.8 (6)	C9—C5—C6—C1	179.4 (4)
C1—C2—C3—C8	178.0 (4)	C9—C5—C6—C10	0.4 (7)
